# MR radiomics to predict microvascular invasion status and biological process in combined hepatocellular carcinoma-cholangiocarcinoma

**DOI:** 10.1186/s13244-024-01741-5

**Published:** 2024-07-09

**Authors:** Yuyao Xiao, Fei Wu, Kai Hou, Fang Wang, Changwu Zhou, Peng Huang, Chun Yang, Mengsu Zeng

**Affiliations:** 1 Department of Radiology, Zhongshan Hospital, Fudan University, Shanghai, China; 2 Shanghai United Imaging Intelligence Co. Ltd, Shanghai, China; 3 Shanghai Institute of Medical Imaging, Shanghai, China; 4 Department of Cancer Center, Zhongshan Hospital, Fudan University, Shanghai, China

**Keywords:** Liver neoplasms, Magnetic resonance imaging, Diagnosis criteria, Prognosis

## Abstract

**Objectives:**

To establish an MRI-based radiomics model for predicting the microvascular invasion (MVI) status of cHCC-CCA and to investigate biological processes underlying the radiomics model.

**Methods:**

The study consisted of a retrospective dataset (82 in the training set, 36 in the validation set) and a prospective dataset (25 patients in the test set) from two hospitals. Based on the training set, logistic regression analyses were employed to develop the clinical-imaging model, while radiomic features were extracted to construct a radiomics model. The diagnosis performance was further validated in the validation and test sets. Prognostic aspects of the radiomics model were investigated using the Kaplan–Meier method and log-rank test. Differential gene expression analysis and gene ontology (GO) analysis were conducted to explore biological processes underlying the radiomics model based on RNA sequencing data.

**Results:**

One hundred forty-three patients (mean age, 56.4 ± 10.5; 114 men) were enrolled, in which 73 (51.0%) were confirmed as MVI-positive. The radiomics model exhibited good performance in predicting MVI status, with the area under the curve of 0.935, 0.873, and 0.779 in training, validation, and test sets, respectively. Overall survival (OS) was significantly different between the predicted MVI-negative and MVI-positive groups (median OS: 25 vs 18 months, *p* = 0.008). Radiogenomic analysis revealed associations between the radiomics model and biological processes involved in regulating the immune response.

**Conclusion:**

A robust MRI-based radiomics model was established for predicting MVI status in cHCC-CCA, in which potential prognostic value and underlying biological processes that regulate immune response were demonstrated.

**Critical relevance statement:**

MVI is a significant manifestation of tumor invasiveness, and the MR-based radiomics model established in our study will facilitate risk stratification. Furthermore, underlying biological processes demonstrated in the radiomics model will offer valuable insights for guiding immunotherapy strategies.

**Key Points:**

- MVI is of prognostic significance in cHCC-CCA, but lacks reliable preoperative assessment.
- The MRI-based radiomics model predicts MVI status effectively in cHCC-CCA.
- The MRI-based radiomics model demonstrated prognostic value and underlying biological processes.
- The radiomics model could guide immunotherapy and risk stratification in cHCC-CCA.

**Graphical Abstract:**

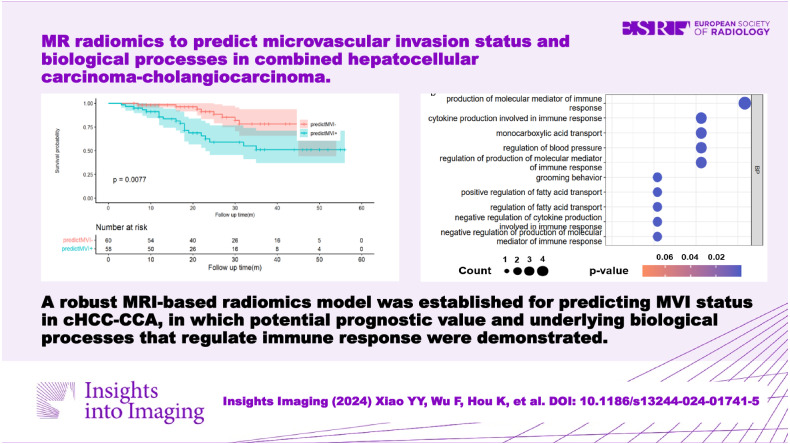

## Introduction

Combined hepatocellular carcinoma-cholangiocarcinoma (cHCC-CCA) is a rare subtype of primary liver cancer (PLC) that contains various proportions of both hepatocytic and biliary components, with an incidence of 0.4–14.2% in PLC [1–4]. Partially due to its rarity and histologic heterogeneity, prognosis and treatment of cHCC-CCA have long been a controversial issue to clarify. Thus, appropriate identification of prognostic factors will facilitate risk stratification and expedite individualized management in cHCC-CCA.

Microvascular invasion (MVI) is a well-defined risk factor in certain tumors [5–8], and the relationship between MVI and the prognosis of cHCC-CCA has been verified by several previous works [9, 10]. Therefore, some researchers, especially radiologists, have paid close attention to the preoperative prediction of MVI in order to function better in clinical practice. Some conventional imaging features and clinical biomarkers, such as the Liver Imaging Reporting and Data System (LI-RADS) categorization, irregular arterial peritumoral enhancement, and serum AFP elevation, have already been determined as significant risk factors for MVI in cHCC-CCA, however, relatively suboptimal interobserver consistency or low sensitivity [10].

Radiomics, as a noninvasive tool to extract quantitative information that is invisible to the naked eye from medical images [11], can potentially capture markers that guide clinical decisions and may be a promising method to predict MVI in cHCC-CCA preoperatively. Moreover, significant differences in gene expression have been demonstrated between MVI-presence and MVI-absence groups in HCC [12–14], and increasing evidence has supported the intimate connection between radiomics features and specific biological portraits [15–18]. Thus, further study is warranted to investigate the biological information of radiomics to validate its clinical value and to further promote clinical transition in cHCC-CCA.

Therefore, the purpose of the present study was to establish a robust MRI-based radiomics model for predicting MVI status of cHCC-CCA, and to investigate the underlying biologic processes of the radiomics model by analyzing RNA sequencing data.

## Materials and methods

This study was approved by the Institutional Review Board and informed consent was required from every enrolled patient.

### Study patients

**Fig. 1:**
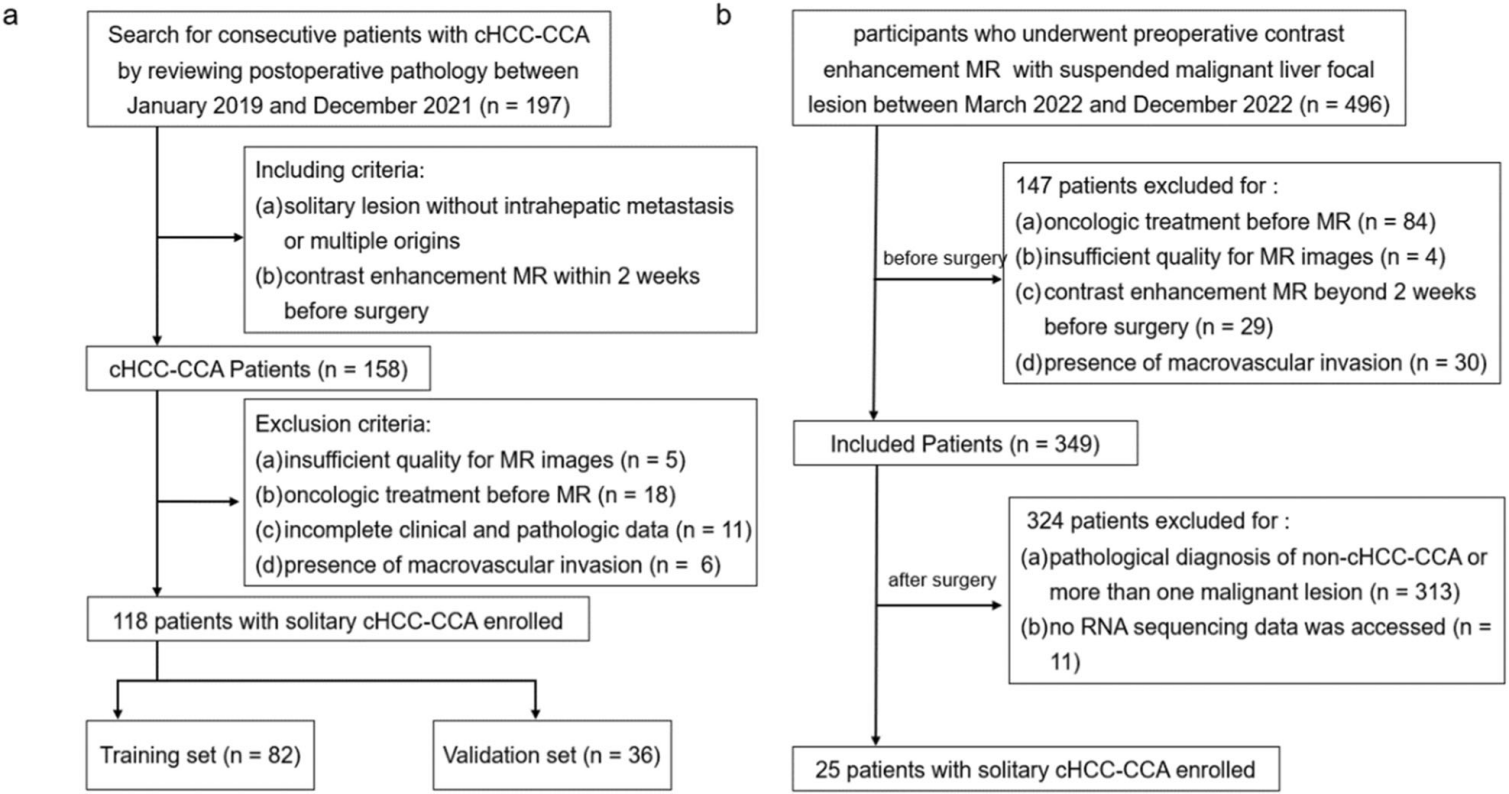
Flowcharts of the patient recruitment process. **a** Training set and validation set. **b** Test set. cHCC-CCA, combined hepatocellular carcinoma-cholangiocarcinoma

For MRI-based radiomics model construction, a total of 158 pathologically confirmed cHCC-CCA patients who underwent surgical resection in Zhongshan Hospital and Shanghai Geriatric Medical Center between January 2019 and December 2021 were retrospectively enrolled by following inclusion criteria: (1) pathologic diagnosis of cHCC-CCA based on the 2019 WHO classification; (2) preoperative contrast-enhanced MRI performed within 2 weeks; and (3) solitary lesion without intrahepatic metastasis or multiple origins. Forty patients were excluded according to the following criteria: (1) any preoperative treatment prior to MRI; (2) insufficient MR image quality; (3) incomplete pathological description data; and (4) presence of macrovascular invasion. Finally, 118 patients were included in our study and were randomly divided into the training set and a validation set in a ratio of 7:3 (Fig. 1a).

For prospective biologic verification of the radiomics model, 25 pathologically confirmed cHCC-CCA patients who underwent surgical resection with RNA sequence data from March 2022 to December 2022 according to the above-mentioned inclusion criteria were enrolled (Fig. 1b), which were named as a test set. This data set was also included in an unpublished paper aiming to explore specific biological portraits of each component in cHCC-CCA.

### Clinicopathological data evaluation

Relevant clinical and pathological data of cHCC-CCA patients were retrieved from medical records retrospectively or prospectively, including age, gender, hepatitis virus infection, tumor size, tumor biomarkers (AFP, CEA, and CA 19-9), and MVI status (MVI + refers to a tumor nest of ≥ 50 suspended tumor cells found within the lumen of the endothelium-lined vessels which is visible only at microscopy). For the evaluation of MVI status, hepatectomy specimens from each patient were viewed microscopically by two pathologists independently.

### MRI technique and conventional MR image analysis

All MR images were acquired via a 1.5-T MR scanner (uMR 560, United Imaging Healthcare). Gadobutrol (Gadavist; Bayer HealthCare) was intravenously administered at a rate of 2 mL/s for a total dose of 0.1 mmol/kg. Routine contrast-enhanced MR imaging protocol included T1-weighted in-phase and out-of-phase sequences, transverse T2-weighted fast spin-echo sequence, diffusion-weighted imaging (DWI) with *b* values of 0 s/mm^2^, 50 s/mm^2^, and 500 s/mm^2^, pre- and post-contrast three-dimensional T1-weighted imaging at arterial phase (20–30 s), portal venous phase (70–90 s), and delayed phase (160–180 s). All detailed parameters of each sequence were previously reported [10].

The MRI images were analyzed by two experienced radiologists, C.Y. and C.W.Z., with 15 years and 14 years of expertise in abdominal imaging analysis, respectively. In case of any discrepancies between the two radiologists, a consensus was achieved through thorough discussion. The evaluation focused on several contrast-enhanced MR features, including enhancement patterns (nonrim arterial phase hyperenhancement (APHE) and rim APHE), washout patterns (nonperipheral washout and peripheral washout), enhancing capsule, delayed central enhancement, and corona enhancement. Additionally, intratumoral hemorrhage, fat deposition, restriction diffusion status (present or absent, rim or nonrim), cholangiectasis, a nodule in nodule architecture, mosaic architecture, and hepatic capsule retraction were also assessed. Targetoid appearance was defined as the presence of any of the following features: rim APHE, peripheral washout, targetoid restriction, and delayed central enhancement. The detailed definitions of these MR features can be found in Table S1.

### Radiomics analysis

A radiologist (Y.Y.X., with 7 years of abdominal imaging analysis experience) performed tumor segmentation by ITK-SNAP software, these segmentation results were checked by a senior radiologist (C.Y., with 15 years of abdominal imaging analysis experience. Volumes of interests were manually delineated on six sequences of pre-T1WI, AP, PVP, DP, T2WI-FS, and DWI with *b* values of 500 s/mm^2^. In addition, MR images of randomly selected 30 lesions were delineated again after 1 month by Y.Y.X. to assess the intra-observer reproducibility, and these 30 MRI images were also delineated by another radiologist (C.W.Z., with 14 years of abdominal imaging analysis experience) independently to evaluate inter-observer reproducibility.

All MR imaging voxels were isotropically resampled to 1 × 1 × 1 mm^3^ to eliminate acquisition-related voxel heterogeneity. Radiomic features were extracted using the uAI Portal (version: 20230715), in which the PyRadiomics tool was embedded, and the *Z*-score method was used to acquire normalized values of the radiomic features.

### Follow-up of recurrence-free survival (RFS) and overall survival (OS)

The RFS time referred to the time interval from surgery to the date of recurrence, death or the last follow-up, while the OS time was defined as the time interval from the surgery to death, the date of the last follow-up or the study end date of July 31, 2023.

### Statistical analysis

Intra- and inter-observer reproducibility was evaluated by using intraclass correlation coefficient (ICC), and radiomic features with ICC ≥ 0.80 in both intra- and inter-observer settings were selected for further analysis. The Spearman correlation analysis, max-relevance and min-redundancy, and least absolute shrinkage and selection operator methods were successively performed to obtain optimal radiomic features. Uni- and multivariate logistic regression analysis were used to develop a clinical-imaging model in the training set. The diagnostic performance parameters of each predictive model, such as the area under the receiver operating characteristic curves (AUC), sensitivity, specificity, accuracy, precision, and F1-score, were calculated. Delong test and McNemar’s test were performed to compare AUCs, accuracy, sensitivity, and specificity, respectively, and the false discovery rate (FDR) was corrected using the Benjamini–Hochberg method. Hosmer–Lemeshow goodness-of-fit test was performed, and calibration curves were then generated. A decision curve was used to evaluate the clinical practicability.

Patients in the prospective RNA sequencing group were divided into low- and high-score groups according to the lower quartile of radiomic score. We then used the DESeq2 package to identify differentially expressed genes (DEGs) with |log2 (fold change)| > 1 and FDR-adjusted *p* < 0.05 between the low- and high-score groups. Statistically significant DEGs were then used to identify distinct gene ontology (GO)-based biological processes. GO highlights the most DEGs and finds the systematic linkages between those genes and biological processes.

Continuous variables were compared using the student *t*-test, ANOVA, Mann–Whitney *U*-test or Kruskal–Wallis *H*-test, and categorical variables were compared using the χ2 test or Fisher’s exact test among different groups. Survival curves were generated and compared by the Kaplan–Meier method and log-rank test. Statistical analyses were performed using R software (version 4.1.1). *p* values less than 0.05 were indicative of a statistical difference.

## Results

### Patient characteristics

A total of 143 patients (mean age, 56.4 ± 10.5; 114 men) were enrolled. Eighty-two and 36 patients were assigned to the training and validation set, and 25 patients were enrolled in the test set. The clinicopathologic characteristics of the three data sets were presented and compared in Table 1. Patients in the validation set had lower rates of hepatitis B infection (63.9%, *p* = 0.015), and patients in the test set exhibited larger tumor size (5 [4–6], *p* = 0.029). The patient characteristics in the training and validation set according to the MVI status are summarized in Table 2. In the training set, 38 patients were assigned to the MVI + group, and these patients were more likely to show larger tumor size (2.6 [1.95–4] vs 3.75 [2.5–6.375], *p* = 0.018), more surface retraction (6.8% vs 31.6%, *p* = 0.004), and more intratumoral hemorrhage (0.0% vs 21.1%, *p* = 0.005). In the validation set, serum AFP level was the only factor that exhibited statistical significance between MVI + and MVI − groups.

**Table 1:** Baseline information of patients with cHCC-CCA in the three data sets

Characteristics	Training set, (*n* = 82)	Validation set, (*n* = 36)	Test set, (*n* = 25)	*p* value
MVI				0.420
Negative	44 (53.7)	15 (41.7)	11 (44.0)	
Positive	38 (46.3)	21 (58.3)	14 (56.0)	
Age (years)^a^	56.7 ± 10.4	54.4 ± 11.7	58.3 ± 8.5	0.344
Gender				0.420
Male	67 (81.7)	26 (72.2)	21 (84.0)	
Female	15 (18.3)	10 (27.8)	4 (16.0)	
Size (cm)^b^	3.25 [2–6]	4.25 [3–6]	5 [4–6]	**0.029**
HBV				**0.015**
Negative	12 (14.6)	13 (36.1)	3 (12.0)	
Positive	70 (85.4)	23 (63.9)	22 (88.0)	
AFP (ng/mL)^b^	22.8 [4.95–141.725]	33.35 [5.875–372.75]	210 [26.8–387]	0.301
CEA (ng/mL)^b^	2.55 [1.6–3.9]	2.5 [1.6–4.025]	2.7 [2–4.6]	0.538
CA199 (U/mL)^b^	18.8 [13.025–33.675]	20.6 [12.075–31.85]	17.9 [9.3–33.4]	0.422
Intratumoral hemorrhage				0.084
Negative	74 (90.2)	27 (75.0)	20 (80.0)	
Positive	8 (9.8)	9 (25.0)	5 (20.0)	
Fat deposition				0.224
Negative	82 (100.0)	35 (97.2)	25 (100.0)	
Positive	0 (0.0)	1 (2.8)	0 (0.0)	
Restricted diffusion				0.818
Negative	6 (7.3)	2 (5.6)	1 (4.0)	
Positive	76 (72.7)	34 (94.4)	24 (96.0)	
Non-rim APHE				0.856
Negative	41 (50.0)	16 (44.4)	12 (48.0)	
Positive	41 (50.0)	20 (55.6)	13 (52.0)	
Rim APHE				0.866
Negative	42 (51.2)	20 (55.6)	14 (56.0)	
Positive	40 (48.8)	16 (44.4)	11 (44.0)	
Non-peripheral washout				0.995
Negative	32 (39.0)	14 (63.6)	10 (40.0)	
Positive	50 (61.0)	22 (26.8)	15 (60.0)	
Peripheral washout				0.923
Negative	77 (93.9)	34 (94.4)	24 (96.0)	
Positive	5 (6.1)	2 (5.6)	1 (4.0)	
Corona enhancement				0.612
Negative	65 (79.3)	26 (72.2)	18 (72.0)	
Positive	17 (20.7)	10 (27.8)	7 (28.0)	
Enhancing capsule				0.597
Negative	36 (43.9)	14 (38.9)	13 (52.0)	
Positive	46 (56.1)	22 (61.1)	12 (48.0)	
Cholangiectasis				0.502
Negative	60 (73.2)	23 (63.9)	19 (76.0)	
Positive	22 (26.8)	13 (36.1)	6 (24.0)	
Surface retraction				0.075
Negative	67 (81.7)	26 (72.2)	15 (60.0)	
Positive	15 (18.3)	10 (27.8)	10 (40.0)	
Nodule in nodule architecture				0.13
Negative	79 (96.3)	31 (86.1)	23 (92.0)	
Positive	3 (3.7)	5 (13.9)	2 (8.0)	
Mosaic architecture				0.384
Negative	55 (67.1)	20 (55.6)	14 (56.0)	
Positive	27 (32.9)	16 (44.4)	11 (44.0)	
Targetoid appearance				0.600
Negative	31 (37.8)	16 (64.0)	12 (48.0)	
Positive	51 (62.2)	20 (36.0)	13 (52.0)	
Targetoid restriction				0.116
Negative	64 (78.0)	30 (83.3)	24 (96.0)	
Positive	18 (22.0)	6 (16.7)	1 (4.0)	
Delayed central enhancement				0.996
Negative	62 (75.6)	27 (75.0)	19 (76.0)	
Positive	20 (24.4)	9 (25.0)	6 (24.0)	
LR categorization				0.408
LR-M	52 (63.4)	20 (55.5)	13 (52.0)	
LR-3	2 (2.4)	0 (0.0)	0 (0.0)	
LR-4	2 (2.4)	0 (0.0)	2 (8.0)	
LR-5	26 (31.7)	16 (44.4)	10 (40.0)	

Bold font indicates *p* values less than 0.05

*cHCC-CCA* combined hepatocellular carcinoma-cholangiocarcinoma, *MVI* microvascular invasion, *HBV* hepatitis B virus, *AFP* alpha-fetoprotein, *CEA* carcinoembryonic antigen, *CA19-9* carbohydrate antigen 19-9, *APHE* arterial phase hyperenhancement, *LR* LI-RADS

^a^ Data are mean ± standard deviation

^b^ Data are median (interquartile range). Except where labeled, data are numbers of patients, with percentages in parentheses

**Table 2:** Patient characteristics in the training set and validation set according to the MVI status

Characteristics	Training set			Validation set		
	MVI −, (*n* = 44)	MVI +, (*n* = 38)	*p* value	MVI −, (*n* = 15)	MVI +, (*n* = 21)	*p* value
Age (years)^a^	55.1 ± 10.4	58.2 ± 9.9	0.166	53.7 ± 12.4	55.0 ± 11.5	0.767
Gender						
Male	40 (90.9)	27 (71.1)	0.974	9 (60.0)	17 (81.0)	0.260
Ffemale	4 (9.1)	11 (28.9)		6 (40.0)	4 (19.0)	
Size (cm)^b^	2.6 [1.95–4]	3.75 [2.5–6.375]	**0.018**	4 [2.6–5.25]	4.5 [3.3–7]	0.351
HBV						
Negative	6 (13.6)	6 (15.8)	0.783	7 (46.7)	6 (28.6)	0.310
Positive	38 (86.4)	32 (84.2)		8 (53.3)	15 (71.4)	
AFP (ng/mL)^b^	21.4 [5.35–110.45]	30.9 [4.05–226.675]	0.827	8.6 [3.7–20.35]	156 [15–1153]	**0.019**
CEA (ng/mL)^b^	2.45 [1.775–3.125]	2.65 [1.525–4.075]	0.625	2.6 [1.7–3.65]	2.5 [1.6–4.1]	0.911
CA199 (U/mL)^b^	20.6 [13.775–35.35]	16.15 [10.75–29.375]	0.530	23 [15.3–38.75]	20.3 [10.2–25.6]	0.427
Intratumoral hemorrhage						
Negative	44 (100.0)	30 (78.9)	**0.005**	13 (86.7)	14 (66.7)	0.252
Positive	0 (0.0)	8 (21.1)		2 (13.3)	7 (33.3)	
Fat deposition						
Negative	44 (100.0)	38 (100.0)	0.508	15 (100.0)	20 (95.2)	1.000
Positive	0 (0.0)	0 (0.0)		0 (0.0)	1 (4.8)	
Restricted diffusion						
Negative	3 (6.8)	3 (7.9)	1.000	2 (13.3)	0 (0.0)	0.167
Positive	41 (93.2)	35 (92.1)		13 (86.7)	21 (100.0)	
Non-rim APHE						
Negative	21 (47.7)	20 (52.6)	0.658	6 (40.0)	10 (47.6)	0.741
Positive	23 (52.3)	18 (47.4)		9 (60.0)	11 (52.4)	
Rim APHE						
Negative	23 (52.3)	19 (50.0)	0.837	9 (60.0)	11 (52.4)	0.741
Positive	21 (47.7)	19 (50.0)		6 (40.0)	10 (47.6)	
Non-peripheral washout						
Negative	18 (40.9)	14 (36.8)	0.707	6 (40.0)	8 (38.1)	1.000
Positive	26 (59.1)	24 (63.2)		9 (60.0)	13 (61.9)	
Peripheral washout						
Negative	41 (93.2)	36 (94.7)	1.000	14 (93.3)	20 (95.2)	1.000
Positive	3 (6.8)	2 (5.3)		1 (6.7)	1 (4.8)	
Corona enhancement						
Negative	38 (86.4)	27 (71.1)	0.088	11 (73.3)	15 (71.4)	1.000
Positive	6 (13.6)	11 (28.9)		4 (26.7)	6 (28.6)	
Enhancing capsule						
Negative	18 (40.9)	18 (47.4)	0.557	5 (33.3)	9 (42.9)	0.732
Positive	26 (59.1)	20 (52.6)		10 (66.7)	12 (57.1)	
Cholangiectasis						
Negative	36 (81.8)	24 (63.2)	0.057	12 (80.0)	11 (52.4)	0.159
Positive	8 (18.2)	14 (36.8)		3 (20.0)	10 (47.6)	
Surface retraction						
Negative	41 (93.2)	26 (68.4)	**0.004**	12 (80.0)	14 (66.7)	0.468
Positive	3 (6.8)	12 (31.6)		3 (20.0)	7 (33.3)	
Nodule in nodule architecture						
Negative	42 (95.5)	37 (97.4)	1.000	12 (80.0)	19 (90.5)	0.630
Positive	2 (4.5)	1 (2.6)		3 (20.0)	2 (9.5)	
Mosaic architecture						
Negative	33 (75.0)	22 (57.9)	0.100	11 (73.3)	9 (42.9)	0.096
Positive	11 (25.0)	16 (42.1)		4 (26.7)	12 (57.1)	
Targetoid appearance						
Negative	18 (40.9)	13 (34.2)	0.533	8 (53.3)	8 (38.1)	0.500
Positive	26 (59.1)	25 (65.8)		7 (46.7)	13 (61.9)	
Targetoid restriction						
Negative	33 (75.0)	31 (81.6)	0.473	10 (66.7)	20 (95.2)	0.063
Positive	11 (25.0)	7 (18.4)		5 (33.3)	1 (4.8)	
Delayed central enhancement						
Negative	34 (77.3)	28 (73.7)	0.706	11 (73.3)	16 (76.2)	1.000
Positive	10 (22.7)	10 (26.3)		4 (26.7)	5 (23.8)	
LR categorization						
LR-M	27 (61.4)	25 (48.1)	0.464	7 (46.7)	13 (61.9)	0.364
LR-3	2 (4.5)	0 (0.0)		0 (0.0)	0 (0.0)	
LR-4	1 (2.3)	1 (2.6)		0 (0.0)	0 (0.0)	
LR-5	14 (31.8)	12 (31.6)		8 (53.3)	8 (38.1)	

Bold font indicates *p* values less than 0.05

*cHCC-CCA* combined hepatocellular carcinoma-cholangiocarcinoma, *MVI* microvascular invasion, *HBV* hepatitis B virus, *AFP* alpha fetoprotein, *CEA* carcinoembryonic antigen, *CA19-9* carbohydrate antigen 19-9, *APHE* arterial phase hyperenhancement, *LR* LI-RADS

^a^ Data are mean ± standard deviation

^b^ Data are median (interquartile range). Except where labeled, data are numbers of patients, with percentages in parentheses

### Construction of prediction model and performance comparison

Tumor size (OR = 2.041, *p* = 0.015) and surface retraction (OR = 4.688, *p* = 0.032) were predictors of MVI status in both univariate and multivariate logistic analysis in the training set, and these two features were then used to construct clinical-imaging model (Table 3), showing unsatisfactory predictive performance, with AUCs in training set and validation set of 0.673 (0.554–0.792) and 0.630 (0.442–0.818), respectively. However, this clinical-imaging model showed a more notable AUC of 0.815 (0.648–0.981), in the test set (Table 4).

**Table 3:** Uni/multivariate logistic regression analysis of MVI status based on clinical and MR imaging features in patients with cHCC-CCA

Feature type	Characteristics	*p* value	OR (95% CI)	*p* value	OR (95% CI)
Clinical features	Gender	0.064	2.951 (0.982–10.125)	0.103	2.713 (0.849–9.765)
	Age (years)	0.170	1.372 (0.882–2.194)		
	**Size (cm)**	**0.010**	**2.11** (**1.25–3.87)**	**0.015**	**2.041 (1.205–3.782)**
	HBV	0.783	0.842 (0.241–2.938)		
	AFP (ng/mL)	0.314	2.558 (0.913–29.603)		
	CEA (ng/mL)	0.192	990608.09 (7.409–NA)		
	CA199 (U/mL)	0.102	2.361 (1.141–9.957)		
Imaging features	Intratumoral hemorrhage	0.990	62399058.099 (0–NA)		
	Restricted diffusion	0.852	0.854 (0.15–4.868)		
	Non-rim APHE	0.658	0.822 (0.342–1.96)		
	Rim APHE	0.837	1.095 (0.458–2.623)		
	Non-peripheral washout	0.707	1.187 (0.487–2.925)		
	Peripheral washout	0.770	0.759 (0.096–4.826)		
	Corona enhancement	0.094	2.58 (0.872–8.298)	0.269	1.955 (0.597–6.693)
	Enhancing capsule	0.557	0.769 (0.318–1.847)		
	Cholangiectasis	0.061	2.625 (0.973–7.491)	0.249	1.896 (0.636–5.758)
	**Surface retraction**	**0.008**	**6.308** (**1.802**–**29.655)**	**0.032**	**4.688 (1.249**–**22.841)**
	Nodule in nodule architecture	0.649	0.568 (0.026–6.157)		
	Mosaic architecture	0.103	2.182 (0.862–5.697)		
	Targetoid appearance	0.533	1.331 (0.543–3.319)		
	LR categorization	0.828	0.974 (0.771–1.231)		
	Targetoid restriction	0.474	0.677 (0.224–1.943)		
	Delayed central enhancement	0.706	1.214 (0.439–3.367)		

Bold font indicates *p* values less than 0.05

*cHCC-CCA* combined hepatocellular carcinoma-cholangiocarcinoma, *MVI* microvascular invasion, *HBV* hepatitis B virus, *AFP* alpha fetoprotein, *CEA* carcinoembryonic antigen, *CA19-9* carbohydrate antigen 19-9, *APHE* arterial phase hyperenhancement, *LR* LI-RADS

**Table 4:** Diagnostic performance of predictive models

Model	Feature number	Group	AUC (95% CI)	Sensitivity	Specificity	Accuracy	Precision	f1 Score	*p* value *
Clinical-imaging model	2	Training set	0.673 (0.554–0.792)	0.474	0.841	0.671	0.720	0.571	**<** **0.001**
		Validation set	0.630 (0.442–0.818)	0.429	0.800	0.583	0.750	0.545	**0.007**
		Test set	0.815 (0.648–0.981)	0.714	0.818	0.760	0.833	0.769	0.781
Radiomics model	26	Training set	0.935 (0.885–0.986)	0.842	0.841	0.841	0.821	0.831	–
		Validation set	0.873 (0.760–0.986)	0.762	0.800	0.778	0.842	0.800	–
		Test set	0.779 (0.580–0.978)	0.786	0.727	0.760	0.786	0.786	–
Clinical-imaging-radiomics model	28	Training set	0.937 (0.887–0.986)	0.868	0.886	0.878	0.868	0.868	0.865
		Validation set	0.873 (0.754–0.992)	0.762	0.800	0.778	0.842	0.800	> 0.999
		Test set	0.786 (0.589–0.983)	0.786	0.818	0.800	0.846	0.815	0.845

Bold font indicates *p* values less than 0.05

*AUC* area under curve

* *p* values were obtained by comparing AUCs of the clinical-imaging model and clinical-imaging-radiomics model with the AUCs of the radiomics model in the training set, validation set, and test set, respectively

A total of 62 significant radiomic features were extracted from six single MR sequences (Table S2), and the prediction performance of each single sequence model in the training and validation set was presented in Table S3 and Fig. S1. Among all single MR sequence models, the pre-TIWI, AP, and PVP models showed the most stable and best diagnostic performance, with a range of AUCs of 0.797–0.958 and 0.759–0.794 in the training set and validation set, respectively. In order to establish a robust multi-sequence radiomics model, the above-mentioned single MR sequence models were combined, referring to the radiomics model. This radiomics model exhibited satisfactory predictive performance with AUCs of 0.935 (0.885–0.986), 0.873 (0.760–0.986), and 0.779 (0.580–0.978) in the training set, validation set, and test set, respectively. Also, this radiomics model consisting of three sequences showed significantly higher AUCs than the clinical-imaging model in the training set (0.935 vs 0.673, *p* < 0.001) and validation set (0.873 vs 0.630, *p* = 0.007), but not in the test set (0.779 vs 0.815, *p* = 0.781). What’s more, the prediction performance of the radiomics model was not inferior to the clinical-imaging-radiomics model in the training set (0.935 vs 0.937, *p* = 0.859), validation set (0.873 vs 0.873, *p* > 0.999), and test set (0.779 vs 0.786, *p* = 0.845). The diagnostic performance of each prediction model is detailed in Table 4 and Fig. S2.

**Fig. 2:**
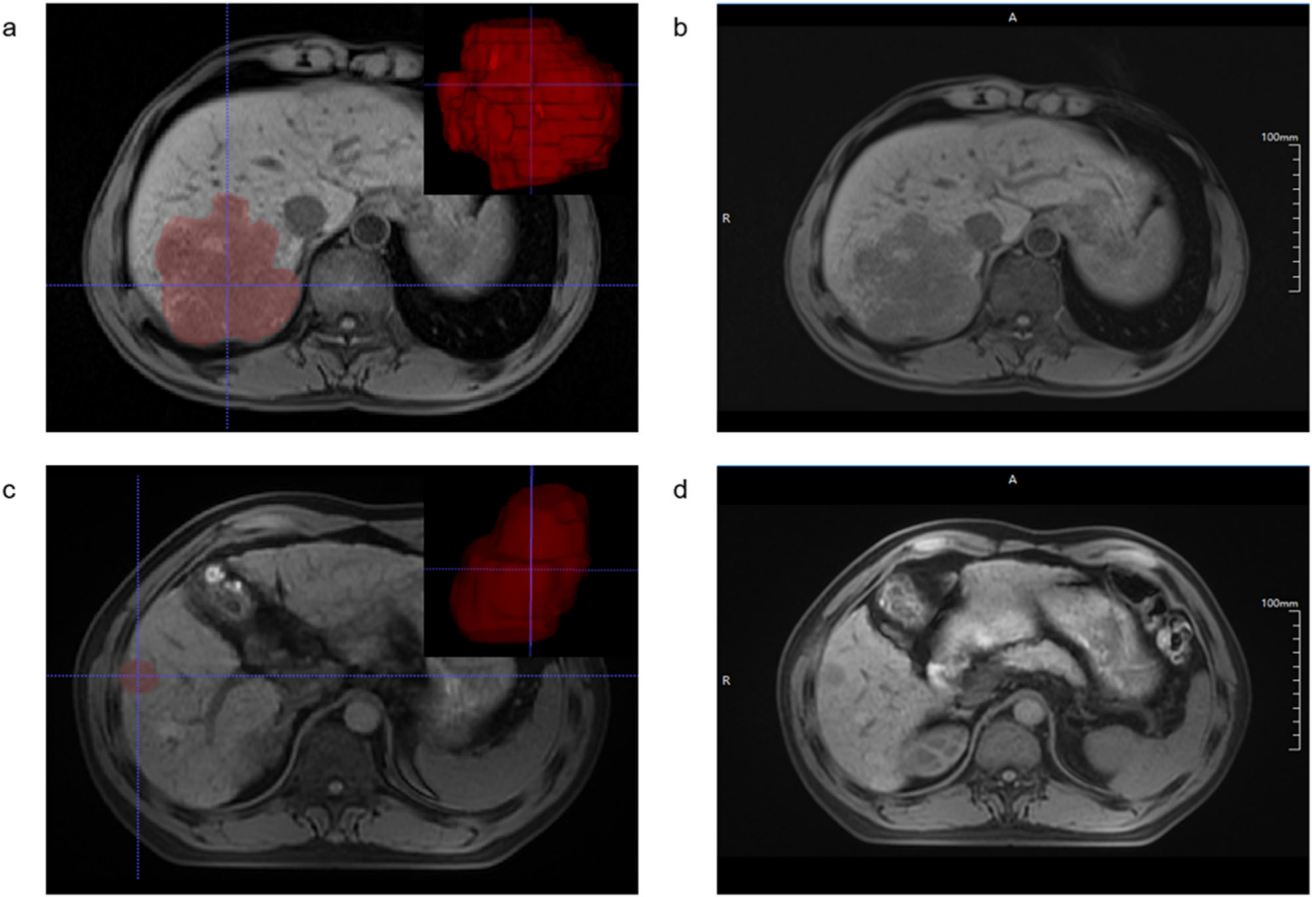
Two examples of applications for MVI status prediction in cHCC-CCA. **a**, **b** Images of a 46-year-old male with a 10.0 cm MVI-positive cHCC-CCA. Based on the radiomics model calculation, the radiomics score for this case is 0.928, and T1-weighted imaging shows homogeneous hypointensity of the lesion, with surface retraction (**b**). The predictive MVI status was positive. **c**, **d** Images of a 51-year-old male with a 2.5 cm MVI-negative cHCC-CCA. Based on the radiomics model calculation, the radiomics score for this case is 0.018, and T1-weighted imaging shows homogeneous hypointensity of the lesion, without surface retraction (**d**). The predictive MVI status was negative

The calibration curve shows the goodness of fit between the predicted MVI status and actual MVI status in three sets (Fig. S3d–f), and all clinical-imaging models, radiomics model, and clinical-imaging-radiomics model showed FDR *p* value of the Hosmer-less how to test higher than 0.05 in all three sets (Table S4). Decision curves of the clinical-imaging model, the radiomics model, and the clinical-imaging-radiomics model in three sets were presented in Fig. S3a–c. Two examples of applications for MVI status prediction in cHCC-CCA using our prediction models are provided in Fig. 2.

### Predictive value of prediction model for survival

All 118 patients in the training and validation sets were followed up after the initial hepatectomy, with a median follow-up time of 21 (range, 3–56) months. The overall recurrence rate was 50.8% (60/118) and the overall death rate was 25.4% (30/118).

**Fig. 3:**
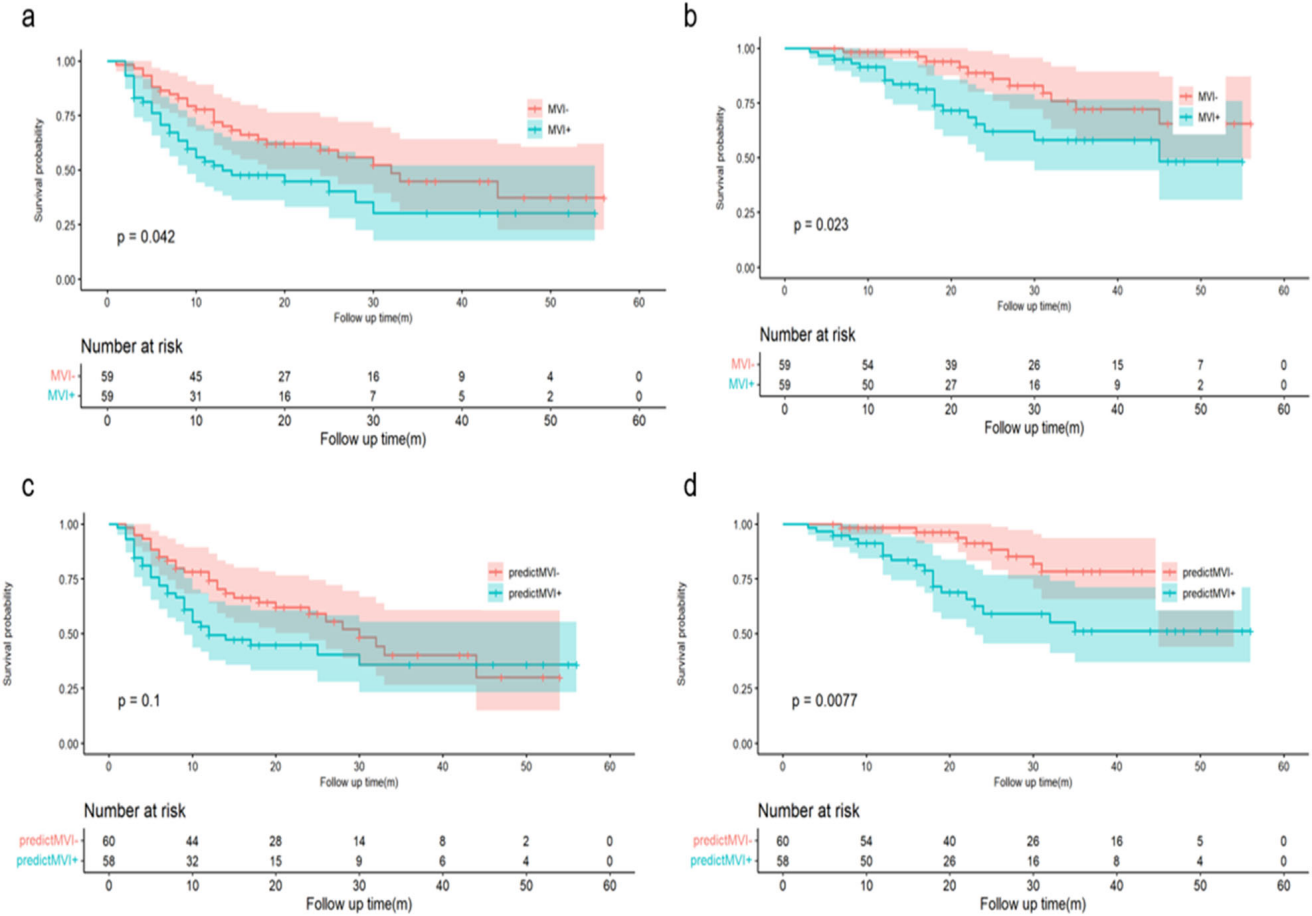
Survival curves according to histological MVI status and predicted MVI status by radiomics model on RFS (**a**, **c**) and OS (**b**, **d**). MVI, microvascular invasion; RFS, recurrence-free survival; OS, overall survival

The median RFS of the patients was 14 (range, 1–56) months, and in particular 10 (range, 2–55) months for MVI + patients and 18 (range, 1–56) months for MVI − patients (log-rank test, *p* = 0.042). In radiomics model, the median RFS was 10.5 (range, 1–56) months for predicted MVI + patients and 18 (range, 2–54) months for predicted MVI − patients with the marginal *p* value of log-rank test of 0.100 (Fig. 3a, c).

The median OS for all patients was 21 (range, 3–56) months, and specifically 18 (range, 3–55) months for those with MVI and 25 (range, 6–56) months for MVI − patients (log-rank test, *p* = 0.023). Similar results were also found in patients stratified by the radiomics model: the median OS was 18 (range, 3–56) months for predicted MVI + patients and 25 (range, 6–56) months for predicted MVI − patients, with the *p* value of log-rank test of 0.008 (Fig. 3b, d).

### Biological processes associated with radiomic score

**Fig. 4:**
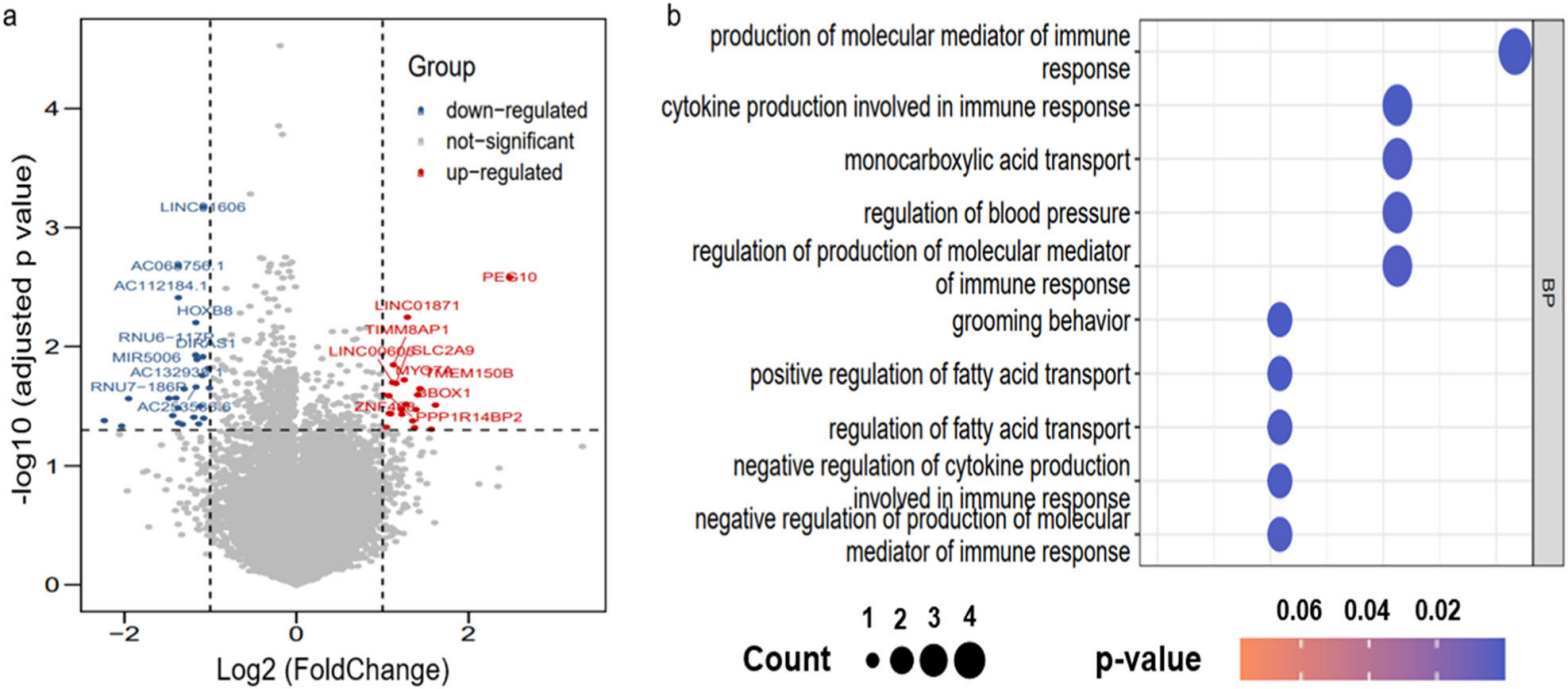
Radiogenomic analysis of biological process associated with the radiomics model. **a** Volcano plot showed the DEGs in the high-score group compared with the low-score group. **b** GO analysis revealed several biological processes associated with radiomics score. GO, gene ontology; BP, biological process

Of the external set with RNA sequencing data, all 25 patients were assigned into low- and high-score groups according to the lower quartile (− 0.976) of radiomic score, by which seven patients were in the low-score group and 18 in the high-score group. Forty-six DEGs were identified to be differentially expressed between the “low-score” and “high-score” groups and were exhibited in Fig. 4a. Further GO analysis was carried out based on these 46 DEGs, and results showed that of the top ten biological processes that were correlated with the radiomics model, five biological processes were implicated in immune response, such as production of molecular mediator of immune response and cytokine production involved in the immune response. *p* value and the number of genes involved in the various biological processes have been listed in Fig. 4b.

## Discussion

Here, we constructed a radiomics model to noninvasively predict MVI status in patients with cHCC-CCA, with AUC of 0.935, 0.873, and 0.779 in the training set, validation set, and test set, respectively. Importantly, our findings based on RNA sequencing data uncovered the underlying biological processes (mainly implicated in immune response) associated with the radiomics model.

This study first established a clinical-imaging model to preoperatively predict the MVI status of cHCC-CCA. The results of univariate and multivariate logistic regression analyses showed that tumor size and surface retraction were independent predictors of MVI status. Tumor size has always been a reliable predictor of MVI status [19–21]: based on the hypothesis of tumor progression [22, 23], the histological grade and invasiveness of tumors increase with increasing tumor size, and thus the risk of MVI also increases with increasing tumor size. Zhou et al [24] also demonstrated that tumor size is an independent predictor of MVI status in cHCC-CCA. In addition, Liao et al [25] explored the application value of clinical and CT imaging features in predicting MVI status in patients with cHCC-CCA, and found that surface retraction is an independent predictor of MVI, which is consistent with our research results. However, the predictive performance of the clinical-imaging model established in this study was not ideal. Therefore, we further established a radiomics model and a clinical-imaging-radiomics model, and compared their predictive performances. The results showed that the predictive performance of the radiomics model was significantly better than that of the clinical-imaging model and was not inferior to that of the clinical-imaging-radiomics model. Based on this, we further explored the prognosis and potential biological significance of the radiomics model.

In recent years, a growing body of evidence has demonstrated that radiomics analysis holds the potential to address diagnostic ambiguity, monitor response to adjuvant therapies, enhance prognostic models, and even visualize the connection between histologic and biologic features of tumors [26–31]. In the current study, we performed a canonical selection of radiomic features for the MVI status prediction model, and then carried out multiple validations to verify its robustness. In the test set, the clinical-imaging model showed statistically equivalent AUC with the radiomics model, as AUCs of the radiomics model were significantly higher than the clinical-imaging model in the training and validation sets, and the relatively small sample size of the test set may account for this. Regardless, our first established radiomics model showed notable and reproducible performance in predicting MVI status in cHCC-CCA, indicating its generalizability in other patient samples.

The prognostic aspects of our radiomics model were also investigated. Histologic MVI of cHCC-CCA has been reported to be a significant prognostic factor of outcome in many studies [9, 10, 32–34] and was also verified by our study. In addition, the radiomics model constructed in our study was not only capable of accurately predicting the MVI status but also has correlations with OS in cHCC-CCA patients. Therefore, our work goes further by showing a radiomics link among MR imaging, MVI status, and clinical outcomes after surgical resection, shedding light on risk stratification and personal management for patients with cHCC-CCA, with enormous clinical translational potential.

One of the primary challenges in radiomic research is the obscurity regarding the underlying biological explanations of radiomic features. Although a fundamental hypothesis behind radiomics is the association between radiomics features and gene profile, no studies have directly investigated this link in cHCC-CCA. In this study, radiogenomic analysis revealed that the radiomics features were associated with several biological processes, most of which were involved in regulating the immune response. The tumor immune microenvironment plays a crucial role in tumor progression and prognosis. A previous study by Nguyen et al [35] determined that, compared with the immune-low subtype cHCC-CCA, the immune-high subtype responded better to immunotherapy and exhibited improved OS; Zheng et al [36] also constructed an immune score based on the densities of immune cells, which holds promise as a valuable prognostic predictor for patients with cHCC-CCA. As the correlation between radiomic score and biological processes involved in regulating immune response was discovered in the present study, the utilization of the radiomics approach to characterize the MVI status will offer valuable insights for selecting patients with cHCC-CCA who may have up- or down-regulated genes associated with regulating immune response, and who may benefit from immunotherapy, thus guiding immunotherapy strategies and risk stratification in cHCC-CCA.

This study has limitations. First, the prediction models were constructed based on retrospectively gathered data, in which selection bias was inevitable. Second, to simplify model construction in the current study, we only enrolled patients with surgically resected single cHCC-CCA, but its generalization would be sacrificed. Third, several studies focusing on the preoperative prediction of MVI in cHCC-CCA indicated that arterial peritumoral enhancement was the significant predictor [10, 37], so radiomics features extracted from the peritumoral area are supposed to be introduced in the future. Finally, the sample size in the present study, especially in the test set, was relatively small, so a multicenter study with a large sample size, for more convincing results and more comprehensive and in-depth transcriptomic analysis, was also warranted in the future.

In conclusion, we established a robust MRI-based radiomics model for predicting MVI status in cHCC-CCA, which demonstrated good diagnostic performance and potential prognostic value. Additionally, the study revealed potential biological processes that regulate immune response underlying the radiomics model, which will offer valuable insights for guiding immunotherapy strategies and risk stratification in cHCC-CCA.

### Supplementary information

ELECTRONIC SUPPLEMENTARY MATERIAL

## Data Availability

The datasets generated or analyzed during this study are available from the corresponding author upon reasonable request.

## Abbreviations

AUC: Area under the curve
cHCC-CCA: Combined hepatocellular carcinoma-cholangiocarcinoma
GO: Gene ontology
MVI: Microvascular invasion
OS: Overall survival
RFS: Recurrence-free survival

## References

[1] World Health Organization (2019) WHO classification of tumours: digestive system tumours. World Health Organization, Geneva. Available via https://publications.iarc.fr/Book-And-Report-Series/Who-Classification-Of-Tumours/Digestive-System-Tumours-2019. Accessed 11 July 2019

[2] Ramai D, Ofosu A, Lai JK, Reddy M, Adler DG (2019). Combined hepatocellular cholangiocarcinoma: a population-based retrospective study. Am J Gastroenterol.

[3] Garancini M, Goffredo P, Pagni F (2014). Combined hepatocellular-cholangiocarcinoma: a population-level analysis of an uncommon primary liver tumor. Liver Transpl.

[4] Beaufrère A, Calderaro J, Paradis V (2021). Combined hepatocellular-cholangiocarcinoma: an update. J Hepatol.

[5] Yuan SX, Yang F, Yang Y (2012). Long noncoding RNA associated with microvascular invasion in hepatocellular carcinoma promotes angiogenesis and serves as a predictor for hepatocellular carcinoma patients’ poor recurrence-free survival after hepatectomy. Hepatology.

[6] Erstad DJ, Tanabe KK (2019). Prognostic and therapeutic implications of microvascular invasion in hepatocellular carcinoma. Ann Surg Oncol.

[7] Hu LS, Weiss M, Popescu I (2019). Impact of microvascular invasion on clinical outcomes after curative-intent resection for intrahepatic cholangiocarcinoma. J Surg Oncol.

[8] Tang Z, Liu WR, Zhou PY (2019). Prognostic value and predication model of microvascular invasion in patients with intrahepatic cholangiocarcinoma. J Cancer.

[9] Wang Y, Zhu GQ, Zhou CW, Li N, Yang C, Zeng MS (2022). Risk stratification of LI-RADS M and LI-RADS 4/5 combined hepatocellular cholangiocarcinoma: prognostic values of MR imaging features and clinicopathological factors. Eur Radiol.

[10] Wang X, Wang W, Ma X (2020). Combined hepatocellular-cholangiocarcinoma: Which preoperative clinical data and conventional MRI characteristics have value for the prediction of microvascular invasion and clinical significance?. Eur Radiol.

[11] Lambin P, Leijenaar RTH, Deist TM (2017). Radiomics: the bridge between medical imaging and personalized medicine. Nat Rev Clin Oncol.

[12] Beaufrère A, Caruso S, Calderaro J (2022). Gene expression signature as a surrogate marker of microvascular invasion on routine hepatocellular carcinoma biopsies. J Hepatol.

[13] Qi LN, Ma L, Wu FX (2021). S100P as a novel biomarker of microvascular invasion and portal vein tumor thrombus in hepatocellular carcinoma. Hepatol Int.

[14] Zhang T, Guo J, Gu J (2019). KIAA0101 is a novel transcriptional target of FoxM1 and is involved in the regulation of hepatocellular carcinoma microvascular invasion by regulating epithelial-mesenchymal transition. J Cancer.

[15] Feng Z, Li H, Liu Q (2023). CT radiomics to predict macrotrabecular-massive subtype and immune status in hepatocellular carcinoma. Radiology.

[16] Aerts HJ, Velazquez ER, Leijenaar RT (2014). Decoding tumour phenotype by noninvasive imaging using a quantitative radiomics approach. Nat Commun.

[17] Yan J, Zhang S, Li KK (2020). Incremental prognostic value and underlying biological pathways of radiomics patterns in medulloblastoma. EBioMedicine.

[18] Li G, Li L, Li Y (2022). An MRI radiomics approach to predict survival and tumour-infiltrating macrophages in gliomas. Brain.

[19] Renzulli M, Brocchi S, Cucchetti A (2016). Can current preoperative imaging be used to detect microvascular invasion of hepatocellular carcinoma?. Radiology.

[20] Taketomi A, Sanefuji K, Soejima Y (2009). Impact of des-gamma-carboxy prothrombin and tumor size on the recurrence of hepatocellular carcinoma after living donor liver transplantation. Transplantation.

[21] Kim SJ, Lee KK, Kim DG (2010). Tumor size predicts the biological behavior and influence of operative modalities in hepatocellular carcinoma. Hepatogastroenterology.

[22] Pawlik TM, Delman KA, Vauthey JN (2005). Tumor size predicts vascular invasion and histologic grade: Implications for selection of surgical treatment for hepatocellular carcinoma. Liver Transpl.

[23] Sakamoto M, Hirohashi S, Shimosato Y (1991). Early stages of multistep hepatocarcinogenesis: adenomatous hyperplasia and early hepatocellular carcinoma. Hum Pathol.

[24] Zhou G, Zhou Y, Xu X (2024). MRI-based radiomics signature: a potential imaging biomarker for prediction of microvascular invasion in combined hepatocellular-cholangiocarcinoma. Abdom Radiol (NY).

[25] Liao ZJ, Lu L, Liu YP (2023). Clinical and DCE-CT signs in predicting microvascular invasion in cHCC-ICC. Cancer Imaging.

[26] Jeong WK, Jamshidi N, Felker ER, Raman SS, Lu DS (2019). Radiomics and radiogenomics of primary liver cancers. Clin Mol Hepatol.

[27] Banerjee S, Wang DS, Kim HJ (2015). A computed tomography radiogenomic biomarker predicts microvascular invasion and clinical outcomes in hepatocellular carcinoma. Hepatology.

[28] Wei J, Jiang H, Gu D (2020). Radiomics in liver diseases: current progress and future opportunities. Liver Int.

[29] Liu X, Khalvati F, Namdar K (2021). Can machine learning radiomics provide pre-operative differentiation of combined hepatocellular cholangiocarcinoma from hepatocellular carcinoma and cholangiocarcinoma to inform optimal treatment planning?. Eur Radiol.

[30] Wang X, Wang S, Yin X, Zheng Y (2022). MRI-based radiomics distinguish different pathological types of hepatocellular carcinoma. Comput Biol Med.

[31] Zhou Y, Zhou G, Zhang J, Xu C, Zhu F, Xu P (2022). DCE-MRI based radiomics nomogram for preoperatively differentiating combined hepatocellular-cholangiocarcinoma from mass-forming intrahepatic cholangiocarcinoma. Eur Radiol.

[32] Chu KJ, Lu CD, Dong H, Fu XH, Zhang HW, Yao XP (2014). Hepatitis B virus-related combined hepatocellular-cholangiocarcinoma: clinicopathological and prognostic analysis of 390 cases. Eur J Gastroenterol Hepatol.

[33] Lee SD, Park SJ, Han SS (2014). Clinicopathological features and prognosis of combined hepatocellular carcinoma and cholangiocarcinoma after surgery. Hepatobiliary Pancreat Dis Int.

[34] Wu Y, Liu H, Zeng J (2022). Development and validation of nomogram to predict very early recurrence of combined hepatocellular-cholangiocarcinoma after hepatic resection: a multi-institutional study. World J Surg Oncol.

[35] Nguyen CT, Caruso S, Maille P (2022). Immune profiling of combined hepatocellular- cholangiocarcinoma reveals distinct subtypes and activation of gene signatures predictive of response to immunotherapy. Clin Cancer Res.

[36] Zheng BH, Ma JQ, Tian LY (2020). The distribution of immune cells within combined hepatocellular carcinoma and cholangiocarcinoma predicts clinical outcome. Clin Transl Med.

[37] Zhang J, Dong W, Li Y, Fu J, Jia N (2023). Prediction of microvascular invasion in combined hepatocellular-cholangiocarcinoma based on preoperative contrast-enhanced CT and clinical data. Eur J Radiol.

