# Supplementary material for: MR radiomics to predict microvascular invasion status and biological process in combined hepatocellular carcinoma-cholangiocarcinoma
Source: Insights Imaging. 2024 Jul 9;15:172. doi: 10.1186/s13244-024-01741-5 (PMC11233482; doi:10.1186/s13244-024-01741-5)
Supplement: Supplementary file 1 — ELECTRONIC SUPPLEMENTARY MATERIAL [file 13244_2024_1741_MOESM1_ESM.pdf]

MR radiomics to predict microvascular invasion status and biological processes in combined hepatocellular carcinoma-cholangiocarcinoma

ELECTRONIC SUPPLEMENTARY MATERIAL

Table S1. Definition of imaging features.

**Imaging features**	**definition**
Intratumoral hemorrhage	a hyperintense area on T1-weighted images, with variable signal intensity on T2-weighted images
Cholangiectasis	intrahepatic duct dilatation within or outside of the lesion
Surface retraction	retraction of hepatic capsular adjacent to the lesion
Non-rim arterial phase hyperenhancement	arterial phase hyperenhancement is not most pronounced in periphery of observation
Rim arterial phase hyperenhancement	arterial phase hyperenhancement is most pronounced in periphery of observation
Corona enhancement	peri-observational enhancement in late arterial phase or early PVP attributable to venous drainage from tumor
Non-peripheral washout	visually assessed temporal reduction in enhancement of an observation relative to composite liver tissue from an earlier to a later phase resulting in nonperipheral hypoenhancement on the portal venous or delayed phases
Peripheral washout	visually assessed temporal reduction in enhancement of an observation relative to composite liver tissue from an earlier to a later phase resulting in peripheral hypoenhancement on the portal venous or delayed phases
enhancing capsule	enhancing rim in portal venous phase or delayed phase
Delayed central enhancement	central area of progressive postarterial phase enhancement.

Table S2. The process of feature selection for radiomic features, and the number of features included in corresponding steps. ICC = intraclass correlation coefficient, MRMR = max-relevance and min-redundancy, LASSO = least absolute shrinkage and selection operator.

Series	Input features	intra-observer	inter-observer	ICC last （both≥0.8）	Spearman rank correlation (p ≤ 0.05)	MRMR	LASSO
pre-T1WI	2264	2188	2219	2162	216	50	5
AP	2264	2213	2240	2201	190	50	11
PVP	2264	2244	2223	2215	173	50	16
DP	2264	2173	2239	2166	272	50	9
DWI	2264	2221	2211	2191	877	50	13
T2WI-FS	2264	2208	2223	2195	204	50	8

Table S3. Diagnostic performance of radiomics features in every single sequences. AUC = area under curve.

Series	feature number	group	AUC (95% CI)	sensitivity	specificity	accuracy	precision	f1Score
AP	11	training set	0.903(0.837-0.969)	0.789	0.841	0.817	0.811	0.800
		validation set	0.759(0.602-0.916)	0.667	0.733	0.694	0.778	0.718
DP	9	training set	0.833(0.744-0.921)	0.684	0.750	0.720	0.703	0.693
		validation set	0.705(0.525-0.885)	0.714	0.667	0.694	0.750	0.732
DWI	13	training set	0.880(0.805-0.955)	0.842	0.818	0.829	0.800	0.821
		validation set	0.702(0.522-0.881)	0.429	0.800	0.583	0.750	0.545
pre-T1WI	5	training set	0.797(0.701-0.893)	0.632	0.841	0.744	0.774	0.696
		validation set	0.794(0.640-0.948)	0.667	0.733	0.694	0.778	0.718
T2WI-FS	8	training set	0.803(0.708-0.898)	0.684	0.795	0.744	0.743	0.712
		validation set	0.641(0.454-0.828)	0.619	0.600	0.611	0.684	0.650
PVP	16	training set	0.958(0.910-1.000)	0.868	0.909	0.890	0.892	0.880
		validation set	0.775(0.619-0.930)	0.857	0.600	0.750	0.750	0.800

Table S4. p values of Hosmer-lemshow test.

Models	p value of Hosmer-lemshow test		
	Train	Validation	Test
clinical-imaging model	0.456	0.241	0.526
radiomics model	0.364	0.307	0.071
clinical-imaging-radiomics model	0.776	0.547	0.123

Figure S1. Comparison of diagnosis performance among different prediction models.

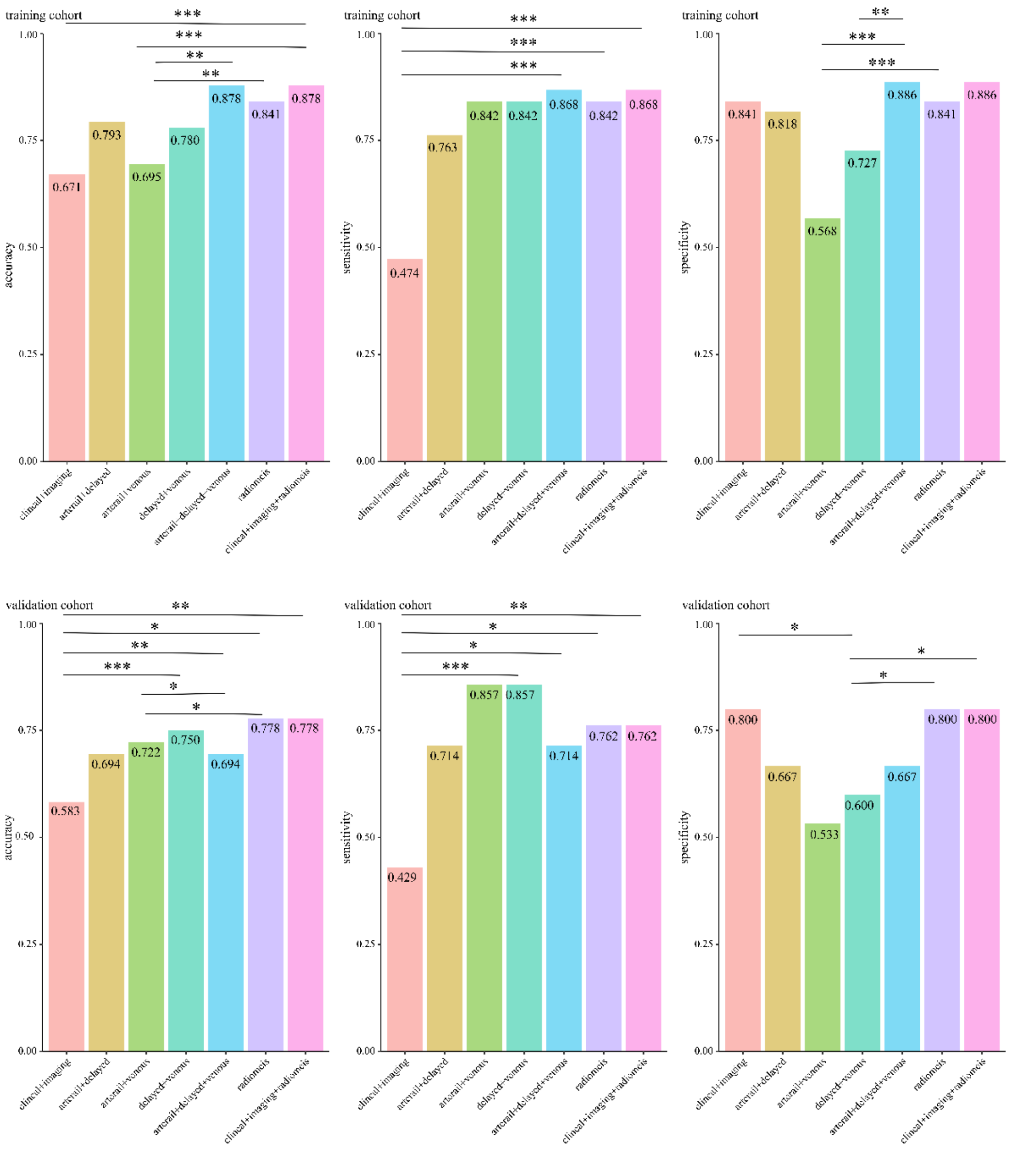

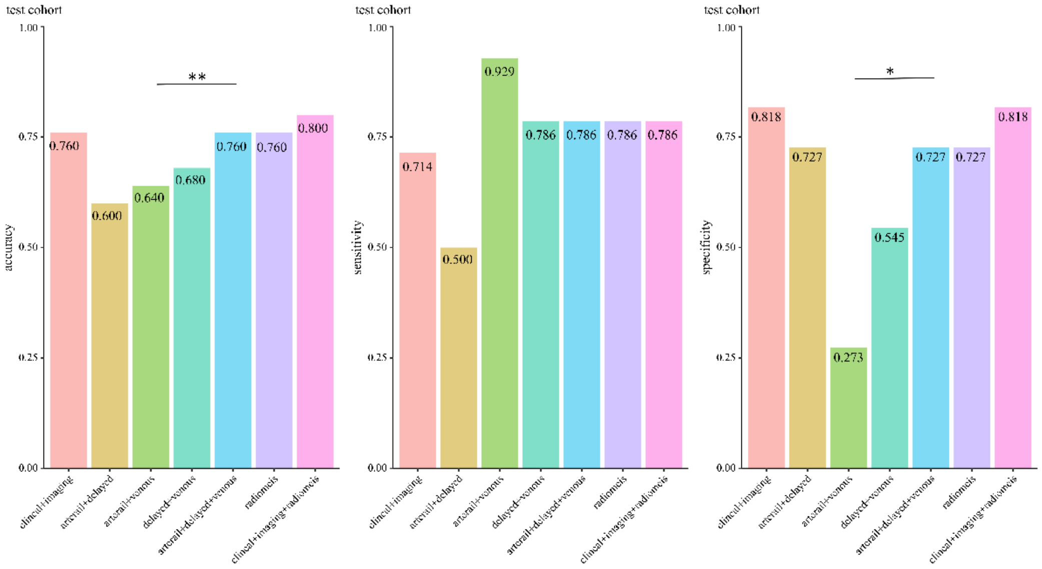
test cohort
accuracy
1.00
0.75
0.50
0.25
0.00
**
0.760
0.600
0.640
0.680
0.760
0.760
0.800
test cohort
sensitivity
1.00
0.75
0.50
0.25
0.00
0.714
0.500
0.929
0.786
0.786
0.786
0.786
test cohort
specificity
1.00
0.75
0.50
0.25
0.00
*
0.818
0.727
0.273
0.545
0.727
0.727
0.818
clincal+imaging
arterail+delayed
arterail+venous
delayed+venous
arterail+delayed+venous
radiomcis
clincal+imaging+radiomcis

Figure S2. AUCs of different prediction models in the three data sets. AUC = area under curve.

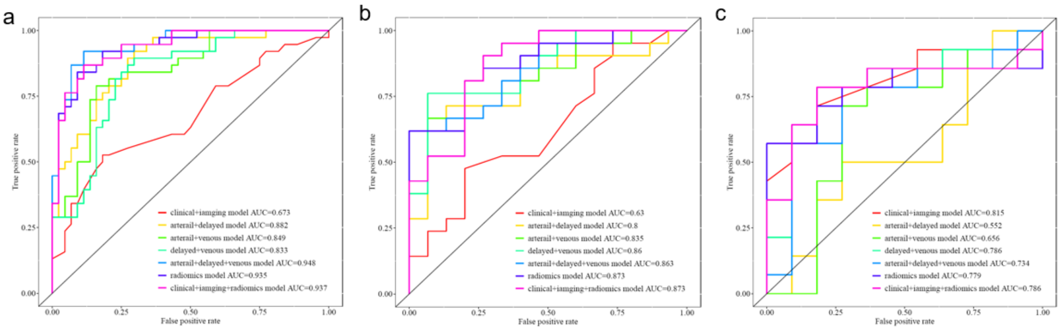

Figure S3. The calibration curves and the decision curve analysis of prediction models in the three data sets.

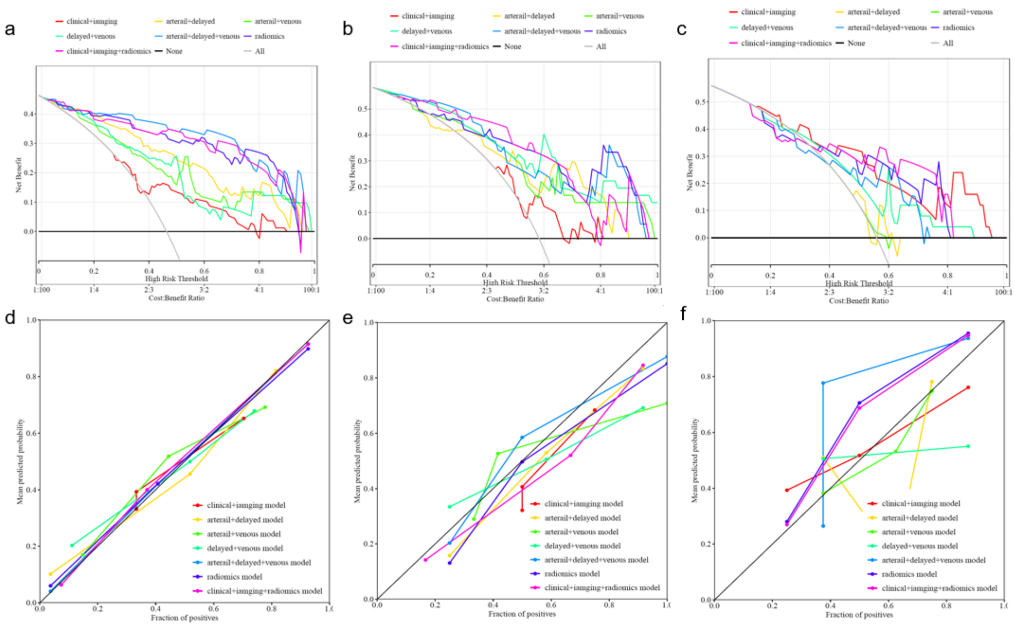

KEGG analysis

Statistically significant differentially expressing genes identified in the main text then underwent Kyoto Encyclopedia of Genes and Genomes (KEGG) analysis to offer a more comprehensive understanding of the immune pathways relevant to this research. According to the results presented in Table S5, we identified two significant pathways that correlated with radiomics model. Mucin-type O-glycans have multiple functions. They bind bacteria and viruses, they function as receptors for carbohydrate binding proteins and are important components of the immune response [1]. Moreover, Xu et al [2] for the first time identified ferroptosis as a novel mechanism of anti-tumor activity, which can be applied in tumor immunotherapy, showing broad clinical application prospects.

Reference

1. Inka Brockhausen and Pablo Argüeso, 3.10 - Mucin-Type O-Glycans: Biosynthesis and Functions, in Comprehensive Glycoscience (Second Edition), J.J. Barchi, J.J. Barchi^Editors. 2021, Elsevier: Oxford. p. 233-252.
2. Xu S, Min J, Wang F. Ferroptosis: an emerging player in immune cells. Sci Bull (Beijing). 2021;66(22):2257-2260.

Table S5. Results of KEGG analysis.

	ID	Description	p value
hsa00512	hsa00512	Mucin type O-glycan biosynthesis	0.04031
hsa04216	hsa04216	Ferroptosis	0.045792
hsa00860	hsa00860	Porphyrin metabolism	0.051245
hsa00514	hsa00514	Other types of O-glycan biosynthesis	0.052332
hsa00520	hsa00520	Amino sugar and nucleotide sugar metabolism	0.054503
